# Deep Hybrid Multimodal Biometric Recognition System Based on Features-Level Deep Fusion of Five Biometric Traits

**DOI:** 10.1155/2023/6443786

**Published:** 2023-07-11

**Authors:** Mohammad Hassan Safavipour, Mohammad Ali Doostari, Hamed Sadjedi

**Affiliations:** ^1^Department of Electrical Engineering, Shahed University, Tehran, Iran; ^2^Department of Computer Engineering, Shahed University, Tehran, Iran

## Abstract

The need for information security and the adoption of the relevant regulations is becoming an overwhelming demand worldwide. As an efficient solution, hybrid multimodal biometric systems utilize fusion to combine multiple biometric traits and sources with improving recognition accuracy, higher security assurance, and to cope with the limitations of the uni-biometric system. In this paper, three strategies for dealing with a feature-level deep fusion of five biometric traits (face, both irises, and two fingerprints) derived from three sources of evidence are proposed and compared. In the first two proposed methodologies, each feature vector is mapped from the feature space into the reproducing kernel Hilbert space (RKHS) separately by selecting the appropriate reproducing kernel. In this higher space, where the result is the conversion of nonlinear relations to linear ones, dimensionality reduction algorithms (KPCA, KLDA) and quaternion-based algorithms (KQPCA, KQPCA) are used for the fusion of the feature vectors. In the third methodology, the fusion of feature spaces based on deep learning is administered by combining feature vectors in in-depth and fully connected layers. The experimental results on 6 databases in the proposed hybrid multibiometric system clearly show the multimodal template obtained from the deep fusion of feature spaces; while being secure against spoof attacks and making the system robust, they can use the low dimensionality of the fused vector to increase the accuracy of a hybrid multimodal biometric system to 100%, showing a significant improvement compared with uni-biometric and other multimodal systems.

## 1. Introduction

**Figure 1:**
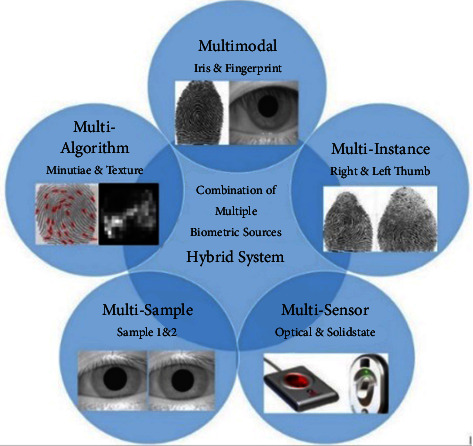
Hybrid system: a combination of multiple biometric sources.

The multibiometric system utilizes fusion to combine multiple biometric sources with improved recognition accuracy [1] while eliminating the limitations of uni-biometric systems relying on a single biometric trait. It is subject to limitations such as noise, poor data quality, nonuniversality, and large variations between users. As far as multiple sources of biometric information are concerned, five possible scenarios exist. Five scenarios can provide biometric information from multiple sources. Multibiometric systems can be classified according to their information sources, such as multisensory, multi-algorithm, multi-instance, multisampling, and multimodal. There are four scenarios in which a single biometric trait (like a fingerprint or an iris) can be used to derive several types of information, while a fifth scenario (which is called a multimodal biometric system) involves the use of several biometric traits (such as fingerprints and iris). The above five scenarios can also be combined into a multibiometric system [2]. These systems are known as hybrid multibiometric systems (Figure 1).

Moreover, to further increase the user authentication's complexity and ensure higher security, more than one trait is combined with each other [3]. Therefore, this paper introduces hybrid multibiometric structures to solve the abovementioned problems. Thanks to stronger reliability, greater applicability, and better security, multimodal biometric systems have been developed for biometric recognition and have attracted more researchers [4].

**Figure 2:**
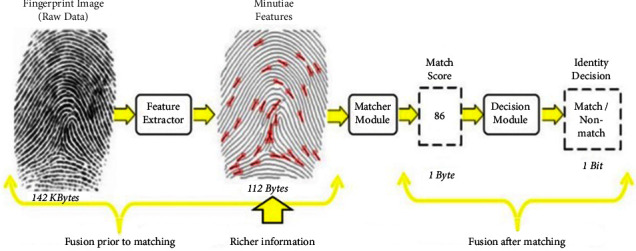
Different levels of fusion in multibiometric.

As shown in Figure 2, there are four levels of biometric data fusion. Raw data will be combined if it occurs at the sensor level. Multimodal systems cannot benefit from this type of fusion; however, it can improve the efficiency of uni-biometric systems. It is possible to combine various biometric features of the same class by using feature-level fusion. It is also possible to combine the scores obtained from multiple classifiers, each of which pertains to a particular biometric. The simplicity and low cost of this method make it ideal for designing multibiometric systems. Decision-level fusion can also occur when several decisions are combined that are each derived from a single biometric system. The effectiveness of decision-level fusion is lower than that of score-level fusion, however. Uni-biometric systems are efficient at recognizing individuals, so both levels can improve limited space usage. A comparison of the four fusion levels reveals that the feature level can extract the maximum discriminative data from the initial feature sets and remove redundant information [4, 5]. Multimodal systems are best devised using feature-level fusion due to the richer information in the feature vectors. There are some applications of machine learning methods in biomedical science [6–9], knowledge science [10, 11], and network and system protection [12–16].

We approach feature-level deep fusion as a design and development technique to achieve a robust and secure hybrid multimodal biometric template. However, it becomes challenging to perform concatenation directly for feature sets with inherent differences in their representation (e.g., IrisCode in Iris and minutiae in fingerprints) [17, 18] Different feature fusion approaches have been explored by several authors [19–21] for fusing other modalities reasonably and effectively. We also proposed three strategies for the feature-level deep fusion of biometric traits.

A feature vector is formed by combining two or more feature vectors in the feature space. As a result, the final vector has a higher detection power than the original vectors. The process of combining feature vectors can be performed by mapping them by selecting the reproducing kernel functions to the reproducing kernel Hilbert space (RKHS) with much higher dimensions, and then fusion of the mapped vectors to RKHS via dimensionality reduction algorithms (KPCA, KLDA) and quaternion-based algorithms (KQPCA, KQLDA).

**Figure 3:**
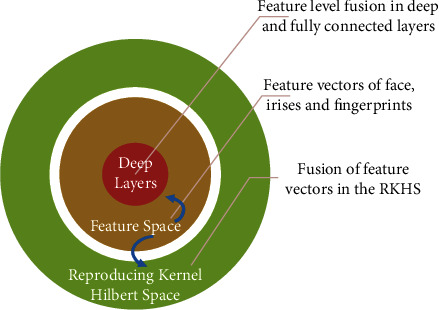
Feature-level deep fusion using mapping in higher space (RKHS) or deep layers.

Or the fusion of feature spaces can be achieved based on deep learning by combining feature vectors in-depth and fully connected layers (Figure 3).

Three strategies are presented here to merge the face, combined iris, and combined fingerprint feature vectors in a hybrid multibiometric recognition system to create a robust and secure multimodal biometric template. We propose and compare dimensionality reduction algorithms (KPCA, KLDA) and quaternion-based algorithms (KQPCA, KQLDA) for reproducing kernel Hilbert space (RKHS) and in-depth learning-based fusion.

As a continuation of this paper, Section 2 introduces the theoretical foundations of the Kernel Method, Hilbert space, RKHS, and Quaternion, as well as deep learning as a method for combining feature vectors in depth and fully connected layers using RKHS. An overview of the design and implementation of the proposed hybrid system is presented in Section 3, which also includes the features extraction, feature-level integration, and classification modules. The results of the analysis and tests are reported in Section 4, and the conclusion is presented in Section 5.

## 2. Theoretical Fundamentals

In this section, to explain the proposed methodology, we present the main theoretical fundamentals we relied upon for our previous works [22, 23].

### 2.1. Kernel Method

The kernel technique is based on the following ideas; Assume that *x*_1_, *x*_2_ ∈*X* ⊆ *R*^*d*^ represent two specimens and *φ*: *X* ⟶ *H* is a feature map of the nonlinear type that transforms each element existing in *X* into a high-dimensional (or even infinite-dimensional) Reproducing Kernel Hilbert Space (RKHS). The interior product between *φ*(*x*_1_) and *φ*(*x*_2_) in the feature spacing *H* may be calculated through the application of a kernel function *k*(*x*_1_, *x*_2_):

(1) $$\begin{array}{c} k\left(x_{1},x_{2}\right)=<\varphi \left(x_{1}\right),\varphi \left(x_{2}\right)>H,\Phi :X⟶H,x⟶\varphi \left(x\right)\text{.} \end{array}$$

Practically, this interior product is obtained from the direct introduction of kernel *k* without having to find the explicit expression *φ*, often called the “kernel trick.” Even though it is effective in learning nonlinear structures, the kernel technique often suffers from scalability drawbacks when dealing with large-scale problems due to extreme space and time complexity [24].

### 2.2. Hilbert Space (*H*)

*H* is a generalized form of Euclidean space in which an extension of vector algebra occurs, where the two-dimensional Euclidean plane and three-dimensional space are extended to finite- or infinite-dimensional spaces. Description and geometric intuition are decisive in numerous attributes of Hilbert's space theoretical framework, especially in infinite-dimensional function spaces. Hilbert space *H* is a complex vector space on which the inner product for each pair of vectors has the following properties:

(2) $$\begin{array}{c} \langle y,x\rangle =\bar{\langle x,y\rangle }\text{,}\\ \langle {ax}_{1}+{bx}_{2},y\rangle =a\langle x_{1},y\rangle +b\langle x_{2},y\rangle \text{,}\\ \langle x,x\rangle >0;x\neq 0,\langle x,x\rangle =0;x=0\text{,}\\ \langle x,{ay}_{1}+{by}_{2}\rangle =\bar{a}\langle x,y_{1}\rangle +\bar{b}\langle x,y_{2}\rangle \end{array}$$

also, *H* with a distance function *d*(*x*, *y*) is a complete metric space such that every Cauchy sequence in *H* has a limit in *H*:

(3) $$\begin{array}{c} d\left(x;\;y\right)=\left\|x-y\right\|=\sqrt{\langle x-y,x-y\rangle }\text{,}\\ d\left(x,z\right)\leq d\left(x,y\right)+d\left(y,z\right)\text{,}\\ \overset{\infty }{\underset{k=0}{\sum }}\left\|uk\right\|\langle \infty \left(CauchySequence-\overset{\infty }{\underset{k=0}{\sum }}uk\right)\text{.} \end{array}$$

In a Hilbert space, the Pythagorean theorem and parallelogram law are exact analogs. However, each element of Hilbert space can be represented based on its coordinates on the axes (normal orthogonal bases). The projection and change of basis in finite-dimensional spaces to Hilbert space are one of the things that show the scope of the applications of Hilbert space.

### 2.3. Reproducing Kernel Hilbert Space (RKHS)

The Hilbert space can be used to extract nonlinearity or higher-order moments from data [25]. For RKHS;

(4) $$\begin{array}{c} x\in X\;,k_{x}\left(y\right)=k\left(x,y\right)\in H\text{,}\\ x\in X\;,f\in H\;;\;f\left(x\right)=\langle f,k_{x}\rangle \text{.} \end{array}$$

#### 2.3.1. Reproducing Property

For any *x* in *X*, a non-zero function *k*_*x*_ exists in *H* so that *f*(*x*)=〈*f*, *k*_*x*_〉_*H*_ for any particular function *f* in *H*, where 〈., .〉*H* represents the inner product of *H*. The above *k*_*x*_ is known as the reproducing kernel of *H* at *x*. Then, the reproducing kernel set {*k*_*x*_}_*x*∈*X*_ is a dense subset of *H*. Set *k*(*x*, *y*)=〈*k*_*y*_, *k*_*x*_〉_*H*_, and it defines a two variable function on *X∗X*.

(5) $$\begin{array}{c} k\left(x,y\right)=k_{y}\left(x\right)=\langle k_{y},k_{x}\rangle \text{,}\\ \left\|k_{x}\right\|={k\left(x,x\right)}^{1/2}={\langle k_{x},k_{x}\rangle }^{1/2}\text{,}\\ k\left(y,x\right)=\langle k_{x},k_{y}\rangle =\langle k_{y},k_{x}\rangle =k\left(x,y\right)\text{.} \end{array}$$

Specifically, *G*=(*k*(*x*, *y*))_*x*,*y*_ may be determined with a self-adjoint matrix in the case where *X* is a finite set, known as the Gram matrix of *H* [26]. Also, it follows from the reproducing property that:

(6) $$\begin{array}{c} \langle \overset{m}{\underset{i=1}{\sum }}a_{i}k\left(∙,x_{i}\right),\overset{n}{\underset{j=1}{\sum }}b_{j}k\left(∙,y_{j}\right)\rangle =\overset{m^{\prime }}{\underset{i=1}{\sum }}\overset{n^{\prime }}{\underset{j=1}{\sum }}a_{i}b_{j}k\left(x_{i},y_{j}\right)x_{m'},\cdots \cdots ,x_{1}\in T,y_{n'},\cdots \cdots ,y_{1}\in T,a_{m'},\cdots \cdots ,a_{1}\in R,b_{n'},\cdots \cdots ,b_{1}\in R\text{,}\\ \overset{n}{\underset{i,j=1}{\sum }}\left(a_{i}a_{j}a_{ij}\right)\rangle 0,A=\left(a_{ij}\right)\rangle 0\text{.} \end{array}$$

The RKHS property is significant in kernel learning because it uniquely determines a reproducing kernel function *k*(*x*,·) in some specific Hilbert space *H*. Practically, the *H* may be abstracted as the image Φ(*x*) with a nonlinear, probably infinite-dimensional, mapping function with the property such as *k*(*x*, *y*)=<*φ*(*x*), *φ*(*y*)>_*H*_ also called as the kernel trick. The kernel trick lets us directly evaluate the inner product developed between *φ*(*x*) and *φ*(*x*′) in *H* without constructing the explicit equation for *φ*. Indeed, some kernels, like the Gaussian kernel, fit into infinite-dimensional feature spaces. There are many kernel-based learning algorithms that calculate only a finite number of data points based on Gram matrices. The Gram matrix *G*∊*R*^*n∗n*^ on a data set *x*_1_,…, *x*_*n*_ is given by *G*_*ij*_=*k*(*x*_*i*_, *x*_*j*_) [27]. Our transfer operators may live in a space of infinite dimensions, but, using Gram matrices deriving from training data, all relevant operations can be performed. Feature spaces can also be infinite-dimensional (e.g., Gaussian kernels) as well as low-dimensional (e.g., polynomial kernels) [28]. In this paper, feature vectors of faces, irises, and fingerprints with appropriate kernel functions are mapped from the feature space to the Hilbert space (with high properties). The change of basis is made in that space to extract more linear relationships.

Gaussian kernel function is as follows:

(7) $$\begin{array}{c} k\left(x.y\right)=\exp\left(-\frac{{\left|x-y\right|}^{2}}{2\sigma^{2}}\right),\left(\sigma \;is\;the\;constant\;parameter\right)\text{.} \end{array}$$

Polynomial Kernel function is as follows:

(8) $$\begin{array}{c} k\left(x.y\right)={\left(xy\right)}^{d},\left(d\;is\;the\;de\;gree\;of\;polynomial\;function\right)\text{.} \end{array}$$

PolyPlus kernel function is as follows:

(9) $$\begin{array}{c} k\left(x.y\right)={\left(xy+1\right)}^{d}\text{.} \end{array}$$

Linear kernel function is as follows:

(10) $$\begin{array}{c} k\left(x.y\right)=\left(x^{\prime }y\right)\text{.} \end{array}$$

Hamming kernel function is as follows:

(11) $$\begin{array}{c} k\left(x.y\right)=1-\frac{1}{mN}\overset{m}{\underset{i=1}{\sum }}\left|x_{i}-y_{i}\right|,\left(m\;is\;the\;number\;of\;image\;pixels\right)\text{.} \end{array}$$

We can calculate the principal component or linear discriminant in space by finding the best kernel function (in RKHS) using high-order correlations of the input pixels. The Matlab code will select the best response based on the mapping of the input image to a higher-order feature space using multiple kernels. Generally, linear kernels, polynomial kernels, Gaussian kernels, and Hamming kernels are the most commonly used kernel functions. The RKHS kernel functions are as follows:

### 2.4. Quaternions

The current section provides a background image of Quaternion algebra and what we call the Quaternion operators used to introduce our models. As a number system in mathematics, the Quaternion number would extend the complex numbers. A Quaternion number is a form of *Q* = *a*.1 + *b*.*i* + *c*.*j* + *dk*, where *a*, *b*, *c*, *d*, are real numbers, and *i*, *j*, and *k* are the fundamental quaternion units. The set ℍ of all Quaternions is a vector space over the real numbers with dimension 4 (*R*^4^) (In comparison, real numbers (ℝ) have dimension 1, complex numbers (ℂ) have dimension 2 (*R*^2^), and octonions have dimension 8 (*R*^8^)). *H* is built in a 4-dimensional vector space, in fact, over the real numbers (*a*, *b*, *c*, *d*), with 1, *i*, *j*, *k* representing the basis, by the component wise add up and the component wise scalar multiplication. The multiplication table of Quaternions defines the multiplication operation for the algebraic system of Quaternions (Table 1) [29].

**Figure 4:**
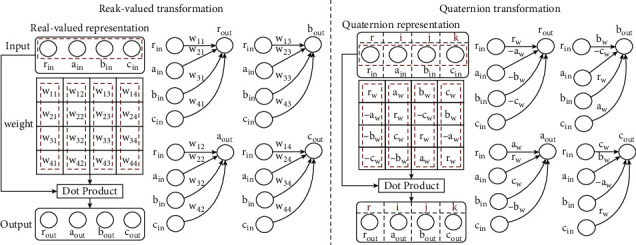
Quaternions versus real-valued transformations (left).

In theorems of Euclidean space, quaternions are advantageous over traditional real-valued approaches for several reasons: (1) quaternions are constructed with one real component and some imaginary components, which would lead to greater expressiveness. (2) The quaternion numbers/vectors are replaced by an application of Hamilton products as a replacement for dot products in Euclidean space, which corresponds best across multiple (inter-latent) quaternions, strengthening their inter-latent relationships, leading to a more expressive model. (3) The Hamilton product is weight sharing, so it would have fewer parameters than a model without it.  Figure 4 illustrates how quaternions are superior to real-valued representations by comparing the transformation procedure with quaternions. Compared with real-valued representations in Euclidean space, quaternions can provide superior inter-dependencies interaction coding with a 75% reduction in parameters [30].

Parallel feature fusion by quaternion may be extended in RKHS to be performed directly with a data-adapted kernel. This corresponds to the parallel fusion of a hyperplane in the probably infinite dimensional space *H*. At the same time, a nonlinear kernel induces the mapped patterns Φ (*x*), the advantage being that using the appropriate kernel in RKHS primarily results in a linear resolution of the nonlinear relationships of the multibiometric features.

**Figure 5:**
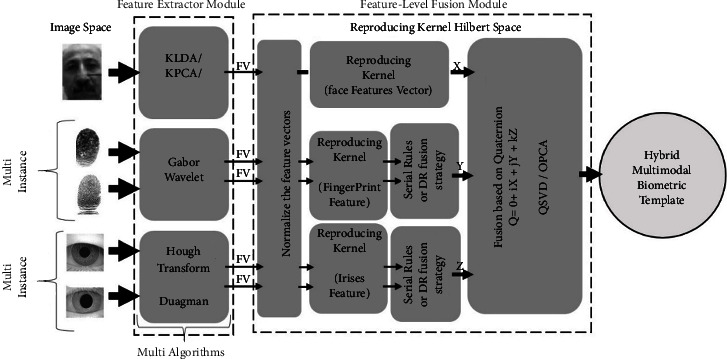
Feature-level fusion methodology based on the parallel fusion using the quaternion in RKHS.

A feature-level fusion module implements the proposed methodology, as shown in Figure 5. Kernel functions are used to map the feature subspaces to the RKHS. A nonlinear kernel mapping with a kernel function that maximizes the interclass scatter while minimizing the intraclass scatter is defined to extract the discriminant information. The RKHS integrates feature vectors using several quaternion-based algorithms, including Quaternion singular value decomposition (QSVD), Quaternion principal component analysis (QPCA), Quaternion linear discriminant analysis (QLDA) [31], and Quaternion locality preserving projection (QLPP). QPCA and QSVD extract the global data from the quaternion division ring, and QLPP extracts the local data, finding the essential manifold construct of quaternion fusion. In addition, QLDA also minimizes conflicts within and between classes and maximizes variance between classes in quaternion fusion feature sets [31]. The result is a quaternion-based fusion in RKHS. The proposed algorithm for achieving a hybrid multimodal template of three feature vectors of face, combined iris, and combined fingerprint in the RKHS by quaternion comprises the following four steps:

- Step 1: Normalize feature vectors
- Step 2: Kernel mapping for the individual input data point *x*_*i*_.
- A nonlinear mapping function *φ* is defined as *φ*: *R*^*D*^ ⟶*H*, *x*  ↦  *φ*(*x*). An implicit high dimensional feature space *F* is formed by mapping the input data feature point *xi* to *RD*. With the nonlinear mapping function *φ* in an RKHS, a kernel function *K*(*x*_*i*_, *x*_*j*_) can be defined as *K*(*x*_*i*_, *x*_*j*_)=〈*φ*(*x*_*i*_), *φ*(*x*_*j*_)〉=*φ*(*x*_*i*_)^*T*^*φ*(*x*_*j*_) where *K* represents a kernel.
- Step 3: Using the serial rule, the two left and right iris feature vectors and the left and suitable index fingerprint feature vectors are combined.
- Step 4: Apply a quaternion-based algorithm to the fusion of features vectors in an RKHS. The three feature vectors *X*, *Y*, and *Z* fill the three imaginary parts of quaternion in the form (*Q*=*Xi*+*Yj*+*Zk*).

According to this, we propose a quaternion-based parallel fusion algorithm in RKHS that model's the fusion of three types of face, iris, and fingerprint biometrics. Unlike the prior works in feature-level fusion, which are based on Euclidean space, our introduced system models the fusion of three biometric feature vectors of a face, combined irises, and combined fingerprints to achieve further efficiency in a reproducing kernel Hilbert space with high dimensional (or even infinite-dimensional) and hypercomplex system (i.e., quaternion space).

### 2.5. Deep Learning

In the previous sections, the theoretical foundations of quaternions and dimensional algorithms in the reproducing kernel Hilbert space (RKHS) were briefly discussed to express one of the proposed models in machine learning aimed at combining the feature vectors of several biometric traits to achieve a hybrid biometric template. Similarly, this section refers to deep learning, which is the construction of a machine learning model applicable to demonstrating a hierarchical display of the data. Deep learning is a potent tool because it manages a lot of data. In deep learning, neural networks with multiple layers are generally referred to as deep neural networks, which illustrate how neural networks with multiple layers can successfully create representational structures. [32–34]. The weights of these networks can be adjusted using feature learning algorithms with and without observers. A convolutional neural network (CNN-based) is one of the most popular deep neural networks. Convolutional, pooling, and fully connected layers make up CNN's architecture. The convolution layer is the backbone of any CNN business model. This layer is where the pixel-by-pixel scan of the images is performed and creates a feature map to define future classifications. By acquiring the overall dimensions of the images, pooling is also known as data sampling. The information on each property from each convolution layer is limited to the essential information. Creating convolution layers and using pooling is continuous; it may be used multiple times. Once the feature analysis has been performed and the calculation time has arrived, the fully connected layer would assign a random weight to the inputs, predicting the appropriate label. The fully connected output layer is the last layer of the CNN model, which contains the results of the tags assigned for the classification and allocates a class to the images.

**Figure 6:**
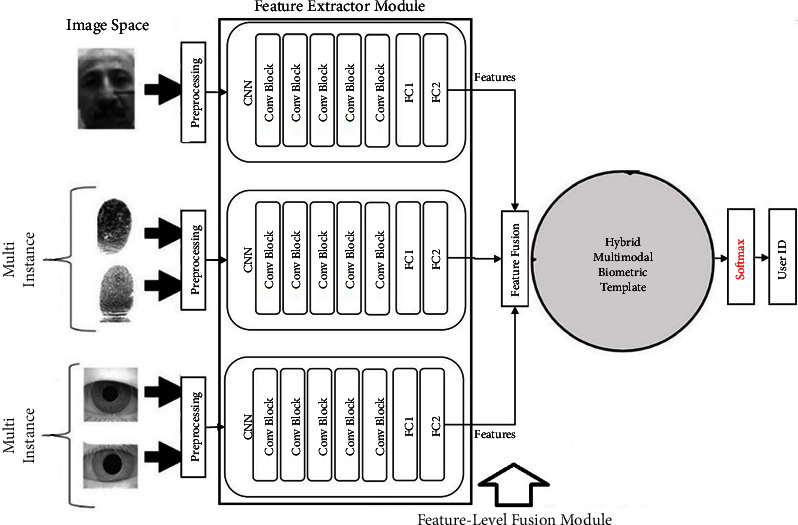
Hybrid multimodal biometric template framework based on feature level fusion in deep layers (CNN).

The filters, therefore, determine the properties through the channeling process, producing an attribute map as their output, as shown in Figure 6. These attribute vectors are placed next to each other and combined to form a hybrid pattern, building a hybrid for our system.

## 3. System Implementation

**Figure 7:**
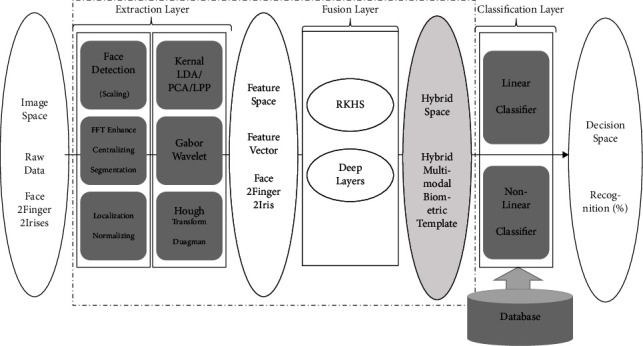
The hybrid multimodal biometric system block diagram.

Figure 7 shows the three main modules of a hybrid multibiometric system: feature extraction, feature fusion, and classification. Additionally, the proposed methodology is implemented in the fusion layer. Modules are divided into subsections.

### 3.1. Feature Extraction Module

An image space is mapped to a feature space in the feature extraction module, which extracts the best features for each biometric trait of the face, iris, and fingerprint. Two methods are generally used for feature extraction of biometric traits in the proposed systems. Applying the first method, a Convolutional Neural Network (CNN) is designed and used to extract face, irises, and fingerprint features, while in the second method, six algorithms are explicitly used to extract each biometric trait: kernel-based algorithms (KPCA & KLDA), which are used to extract face features, Log-Gabor filter, applicable for extracting fingerprint features, and Daugman and Hough transform algorithms that are used to extract right and left iris features. This section briefly explains the algorithm for extracting faces, irises, and fingerprint feature vectors.

#### 3.1.1. Face, Iris, and Fingerprint Features Extraction

The unimodal iris, face, and fingerprint feature extraction algorithms used in our previous work were reused in the proposed hybrid multimodal biometric system using the three traits (iris, face, and fingerprint).

*(1) Face Feature Extraction.* There are three types of face feature extraction methods: model-based, template-based, and appearance-based. Several appearance-based methods are available, including principal component analysis (PCA), linear discriminant analysis (LDA), locality preserving projections (LPP), local binary patterns (LBP), and discrete cosine transforms (DCTs). Nonlinear methods include kernel principal component analysis, kernel linear discriminant analysis, and kernel locality preserving projections. A face experiences extensive intrinsic (i.e., age wrinkles, facial expression variations) and exterior changes (i.e., occlusions, gestures, and lighting variations) changes in the real world. Because of high cost and complex calculations, it may be challenging and difficult to provide many experimental face photos. Accordingly, face recognition is often a nonlinear issue due to the small-scale photos' complicatedness, number, and dimensionality. That explains the importance of developing algorithms with useful features and nonlinear scales for describing the nonlinear relationsships between face samples. Considering that the kernel method can effectively register nonlinear similarities between samples, kernel-based face feature extraction methods have been introduced to develop linear algorithms in which appropriate kernel functions are used to map the samples implicitly into a new feature space with higher dimensionalities. Then, in this new feature space, nonlinear relations become linear, and the distance metrics are trained for the desired use. Of course, unlike the excellent performance of kernel functions in various algorithms, one key matter in the algorithms is to select the appropriate kernel or parameters for a specific kernel [35]. This paper uses KPCA [36] and KLDA [37] to extract face features.

*(2) Iris Feature Extraction*. The iris recognition system is an accurate and reliable biometric technology. The Daugman algorithm [38] and the Hough transform [39] extract iris features. The iris feature extraction algorithm may be summarized in three steps:

(1) Identifying the iris boundaries in an eye image is the first and most vital step in iris recognition. In the Duagman algorithm, (*x*, *y*) denotes the iris image, *r* denotes the radius range for searching in the image, and *G*(*r*) represents the Gaussian smoothing function that starts searching from the pupil to distinguish changes in the maximum pixel values (partial derivative). Two circles represent the eye in the Hough transform, with (*xi*, *yi*) denoting the center coordinates and *r* denoting the radius.
(2) A rectangular block is created by normalizing images segregated from circles into equal dimensions.
(3) An iris feature is extracted using the 1D Log-Gabor filter on a normalized image to display information about the tissue of the iris. This filter is a logarithmic-scale Gabor function, and the frequency of the Log-Gabor filter is provided for in the following formula, where *f*0 denotes the frequency center and *σ* is the filter bandwidth:
  (12) $$\begin{array}{c} G\left(f\right)=\exp\;\left(\frac{-{\left(\log\;\left(f/f_{0}\right)\right)}^{2}}{{2\left(\log\;\left(\sigma /f_{0}\right)\right)}^{2}}\right)\text{.} \end{array}$$

A 9600-bit code is used to process the features of the iris, while a 9600-bit code is used to process the upper and lower eyelashes.

*(3) Fingerprint Feature Extraction.* Fingerprint verification techniques can be categorized as based on correlation, based on ridge features, and based on minutiae. The ridges and valleys pattern can also be considered an oriented texture. The minutiae pattern is unique, but many factors affect the system performance, such as noise and distortion occurring while acquiring the image [40]. To solve the problems with fingerprint matching algorithms, new representations and techniques of fingerprint images have been proposed recently [41]. False fingerprints made from unique materials can attack fingerprint identification systems. A fingerprint liveness detection algorithm (FLD) has been developed to enhance fingerprint identification systems' security. Image quality, sweat pores, perspiration, skin deformation, and texture features are the five categories of software-based FLD methods. The first four FLD methods have a poor user experience as they require two or more images to compare. Using texture features, the first four methods are solved by analyzing fine texture information individually and measuring using just one image. Visual traits such as texture reflect the surface structure's arrangement properties and describe an image's homogeneity phenomenon. For FLD, fingerprint texture information can be used to determine the morphology, smoothness, and orientation of authentic and fake fingerprints. [42].

The fingerprint feature space is based on the fingerprint texture feature. In fingerprint feature extraction, methods such as minutiae matching, short time Fourier transform (STFT), and Gabor filter bank [43] are used. Gabor filter banks are commonly used to extract features.

There are four major steps to the fingerprint feature extraction algorithm after improving the fingerprint image:

1. Identifying the target area and the reference point
2. A reference point is used to segment the target area
3. The Gabor filter bank can be used to filter the target area in six or eight different directions
4. Calculate the absolute standard deviation of each segment to generate a feature vector [44].

#### 3.1.2. CNN-Based Feature Extraction

In the VGG-16 architecture [45], CNNs comprise convolutional layers, pooling layers, and all-connected layers. An input image is processed by a convolutional layer using a sliding window technique. A feature map is produced by convolutioning the original image, in which various features are captured, including edges, corners, etc., from the original image. This allows different types of filters to produce different feature maps. Afterward, the production of a convolution layer, usually a nonlinear activation function (e.g., Rectified Linear Unit (ReLU), is introduced elementwise, so a rectified feature map can be generated. This ReLU, when activated, would replace the total of negative pixels with values equal to zero in a feature map. To decrease the dimensionality of the rectified feature map, a pooling layer should be used. By pooling all the pixels in a feature map's local neighborhoods, the pooling retains all the significant information on the map.

**Figure 8:**
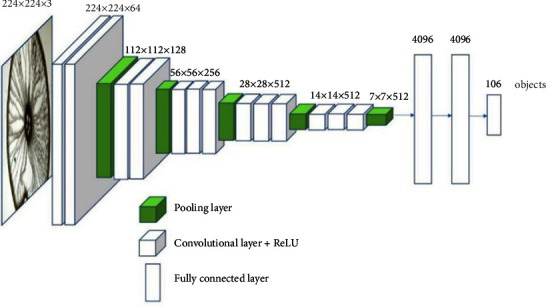
Feature extraction through CNN-based fusion-VGG-16 [46].

Therefore, the feature map is equivariant of scale and translation [46]. Following the nonlinear activation and pooling of layers, the convolutional layers are sequenced; CNNs have one or more fully connected layers, in which all neurons are connected to all neurons in the subsequent layer, so that the first fully connected layer is coupled to the last minimized feature map. By utilizing the fully connected layers, the dimensionality could be further reduced and nonlinear dependencies could be captured. There are an equal number of output neurons in the last fully connected layer compared to the targeted classes. This layer uses a function called “softmax.” Several pretrained CNN architectures are currently available, including the VGG-16 [46]. The VGG-16 network provides outstanding efficiency regarding the ImageNet competition, in which the network is trained with countless images in one thousand categories. Moreover, the VGG-16 was utilized with proper results in our previous work, that is, the Faster R-CNN paper, giving us the impetus to reuse it in the current study. This VGG-16 has thirteen layers of convolutions + reLU, five layers of pooling, and three layers of fully connected layers [46] (see Figure 8).

### 3.2. Feature Fusion Module

A single vector as a hybrid multimodal template is the output of the feature fusion module, which is obtained by combining the three vectors of face, iris, and fingerprint features. This vector has more differential power than the output feature vectors of the feature extraction module. Figure 2 illustrates the feature space that contains the richest data. It means feature vectors are better quantitatively and qualitatively than other levels in terms of information. In two aspects, data fusion in image space is essential. The first is that it derives discriminant information from the original set of features; the second is that it can remove unnecessary and repetitive information because of the correlation between separate sets of features. In other words, feature fusion would produce the best vector to create maximum distinction and have the minimum dimensions for the system to make the best decision [1].

The process of vector fusion in the feature space can be implemented in three ways: “series or parallel combination” [2], “feature extraction algorithm, or dimensionality reduction methods” [47], or “binary feature fusion” [2]. The combination of feature vectors constitutes one of the three feature-level fusion strategies. There are three principal modes for combining feature vectors: serial rule, weighted sum rule [48], and parallel fusion. The first one consumes abundant computational resources while adopting the last one; the weight selection is problematic. Parallel fusion was proposed by Yang et al. [49]. It avoids the huge amount of computation in the serial rule and the choice of weights in the weighted sum rule. But this method takes two features as the real and imaginary parts of a complex vector, and it can only fuse two single feature modalities or one modality with two types of the feature. Often, multimodal biometric recognition must benefit from more modalities or features for higher efficiency.

For this reason, this paper proposes the parallel fusion of three or more feature vectors using the Quaternion algorithm. Of course, for better performance, before the Quaternion fusion, feature vectors are mapped into the Hilbert space using reproducing kernels to extract more nonlinear relationships. As shown in Figure 5, the features extracted from the 3 traits underwent fusion to generate new features representing the user. Because of the fusion strategy discussed above, the model could recognize the combined features throughout the training phase. As a result, a fusion of the second fully connected layer output of the face, iris, and finger vein CNNs is performed. By combining three CNN models, the vectors resulting from the 2nd fully connected layer become a single vector, defined as follows:

(13) $$\begin{array}{c} X=xr\left|xf\right|xv\text{.} \end{array}$$

An iris image contains *xr* characteristics, a face image contains *xf* characteristics, and a finger vein image contains *xv* characteristics. To identify the person, the resulting vector *X* is then input into the softmax classifier to classify the image based on the similarity score [19].

### 3.3. Classification Module

**Figure 9:**
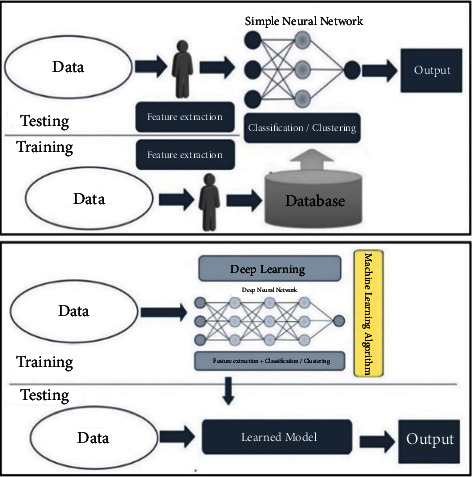
Deep hybrid multimodal biometric training and testing systems.

As shown in Figure 9, training and testing of the hybrid system are proposed in two ways.

As part of the testing phase, the same parameters are applied to new data to determine the level of distinction between consequential feature vectors. A comparison is then made between the classification results and a favorable target function to determine the efficiency of the system. Obtaining the weights of each neuron in a neural network is similar to determining the effectiveness of the neural network.

In deep learning, a trained model is obtained from the training data. The testing data are entered into this model (which can be a deep neural network and includes all the extraction, fusion, and classification modules). The result would be accessible from the output. Using the other method, as explained in previous sections, the features of the face, iris, and fingerprint images are extracted first. Then, after normalization by mapping feature vectors to the RKHS, quaternion-based algorithms are used to fuse and store a multimodal template of biometrics representing each class in the database. Finally, in the recognition phase, the classification module compares the new hybrid biometric template obtained from the previous modules (extractor and feature fusion) with the hybrid modules previously stored in the database in the enrolment phase to determine its class based on further similarity (or shorter distance) between the new template and the stored template. In order for a system to be efficient, it is imperative that the classification module perform well. The results of nine classifiers have been evaluated in this article, as shown in Figure 8. In addition to distance function classifiers (Euclidean, Manhatan, Angle, Mahalanobis), probabilistic neural network classifiers (PNNs), radial basis function neural networks (RBFs), k-nearest neighbor classifiers, kernel support vector machines (KSVMs), and Gaussian classifiers, these classifications include k-nearest neighbor classifiers.

## 4. Evaluation Metrics

It is essential to evaluate a model before developing a machine learning or deep learning model for practical applications. A trained model was used to classify the test images after preprocessing, training, and validation. It is proposed to measure the performance of uni-biometric, multi-instance, multialgorithm, and hybrid multimodal biometric systems [22, 34] by looking at performance metrics such as recognition accuracy, receiver operating characteristics (ROC) curves, AUCs (area under ROC curves), sensitivity, specificity, and efficiency [22, 34]. In Table 2, true positives (TP) represent correct positive example assignments, false negatives (FN) indicate incorrect negative example assignments to positive classes, false positives (FP) indicate incorrect positive example assignments to negative classes, and true negatives (TN) indicate correct negative example assignments.

- Sensitivity: This is also known as the recall rate or the true positive rate, and is a measure of how well the classifier performs. Calculates the percentage of positives that are correctly identified.
- Specificity: It is the percentage of samples that test negative with the test on an actually negative issue, which is often referred to as the specificity of a test. The rate at which negatives are correctly identified, in other words. All healthy individuals are identified as negative for a given condition by a test, for example. Specificity measures are based on the percentage of accurately diagnosed healthy individuals across all healthy groups.
- Accuracy: The accuracy metric measures how many samples were correctly identified. Measurements that are close to a specific value
- Precision: In the precision metric, relevant issues are calculated as a percentage. The measure of how close the measurements are to each other. Classifiers are evaluated according to their ability to reject irrelevant subjects. Because of the recall metric, relevant subjects are found in many samples. During the classification process, it considers how well the classifier presents all the relevant subjects.
- Positive likelihood ratio (PLR): likelihood ratio positive, the likelihood ratio for positive results is the probability of a test case with the condition testing positive divided by the probability of a test case that does not have the need to test positive.
- Negative likelihood ratio (NLR): likelihood ratio negative or likelihood ratio for negative results is the probability of a test case with the condition testing negative divided by the probability of a test case that does not need to test negative.

Receiver operating characteristic curves are developed by plotting true positive rates (TPR) against false positive rates (FPR) with different thresholds. The following shows this clearly: the below curve area can be computed by (the integral boundaries are inevitably inversed because the large threshold *T* value is lower on the *x*-axis):

(14) $$\begin{array}{c} AUC=\overset{1}{\underset{x=0}{\int }}TPR\left({FPR}^{-1}\left(x\right)\right)dx\text{.} \end{array}$$

ROC curves and verification performance alone cannot validate a multibiometric system's performance. Research on in-person authentication, which analyzes whether two models differ statistically significantly from one another, is essential but has received insufficient attention. As a result, Bengio and Mariéthoz [50] presented a confidence interval (CI) and half total error rate (HTER) statistic. Using these two parameters, this study tests our method. HTER is computed as follows:

(15) $$\begin{array}{c} HTER=\frac{FPR+FNR}{2}\text{.} \end{array}$$

To compute CI around HTER, we look for the bound *σ* × *zα*/2. Here, *σ* And *zα*/2 is defined as:

(16) $$\begin{array}{c} \sigma =\sqrt{\frac{FPR.TNR}{4.NI}+\frac{FNR.TPR}{4.NG}}\text{,}\\ \frac{z\alpha }{2}=\left\{\begin{array}{c} 1.645for90\%CI\\ 1.960for95\%CI\\ 2.576for99\%CI \end{array}\right. \end{array}$$

NG and NI indicate the number of intraclass comparisons and interclass comparisons [51]. For the introduced system, performance parameters such as recognition accuracy, ROC curve, AUC, sensitivity, specificity, and efficiency are presented. 100 classes are considered for system training and testing in these tests. For this purpose, the faces, right and left irises, and the right and left index fingerprints of 100 persons registered in the databases above were selected to extract their feature vectors. Eighty percent of the images of each person (class) are used in training and the remaining twenty percent in testing.

## 5. Experimental Results Analysis and Discussions

A multimodal biometric device's efficiency is greatly influenced by the fusion methodology adopted. Because of the high quality and quantity of information in the feature space, feature-level fusion is more effective than fusion at other levels. Therefore, the feature-level fusion technique was the most stable. We use a fusion of feature vectors to convert five feature vectors into a single vector to achieve a multimodal biometric template that is robust, secure and has higher detection power than the original vectors. However, the feature set derived from multiple biometric features and used for multimodal device design could be inconsistent [52]. This is the challenge we face in the fusion of different feature spaces. The fusion of feature space is done through one of the three processes “series or parallel combination,” “feature extraction algorithm or dimensionality reduction methods,” or “binary feature fusion.” In this article, we propose three fusion methodologies to avoid the inconsistency problems of the feature space of five biometric traits derived from three sources of evidence. This section compares the results of strategies of serial combination, dimensionality reduction, parallel fusion, and CNN-based fusion in multi-instance fingerprint, multialgorithm iris, and deep hybrid multimodal recognition systems. A deep hybrid multimodal biometric recognition system uses deep learning and quaternion-based and dimensionality reduction algorithms in the RKHS for an effective fusion strategy and accurate biometric recognition. Feature-level deep fusion is performed using mapping in higher space (RKHS) or deep layers; the process of combining feature vectors can be performed by mapping them by selecting the reproducing kernel functions to the RKHS with much higher dimensions and then fusion of the mapped vectors to the RKHS via dimensionality reduction algorithms. Also, parallel feature fusion by quaternion may be extended in RKHS to be performed directly with a data adapted kernel. The advantage is that using the appropriate kernel in RKHS largely results in a linear resolution of the nonlinear relationships of the multibiometric features. The deep learning-based fusion model in Figure 6 with fully connected layers shows the architecture of the multimodal CNN network for deep feature fusion of the features of the face, irises, and fingerprints.

We perform experiments on six databases, including the two face databases (FERET [53] and Shahed-University (gathered at Shahed University, Tehran, Iran)) [23], CASIA iris databases (right and left irises) [54], and both right and left index fingerprint databases of Shahed-University.

First, we present results from multibiometric recognition systems using iris, fingerprint, and face data separately with corresponding classifiers. A multialgorithm and multi-instance recognition system examines the fusion of two fingerprints and both right and left irises. As a final illustration, the recognition results of the hybrid multimodal biometric system are illustrated using the same classifiers that were used to combine the features of the face, two irises, and two index fingerprints.

### 5.1. Uni-Biometric Recognition Systems

**Figure 10:**
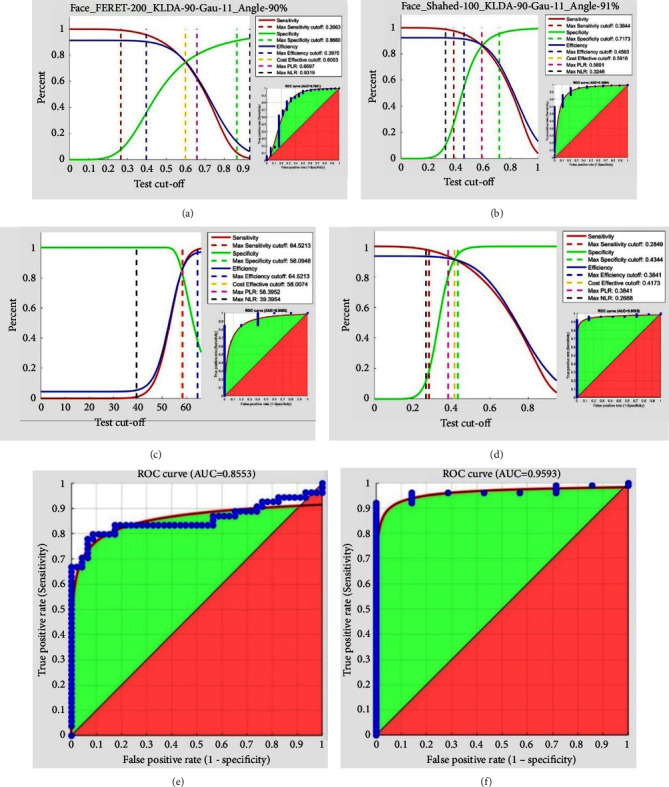
The ROC curve and uni-biometric recognition system analysis on various databases, including (a) FERET face database (AUC:0.7881), (b) shahed face database (AUC:0.9094), (c) right iris CASIA database (daugman algorithm.) (AUC: 8892), (d) left iris CASIA database (hough tra.) (AUC: 0.9593), (e) shahed right index fingerprint database (AUC = 0.8553), and (f) shahed left index fingerprint database(AUC = 0.9593).

Figure 10 compares the AUC with the CNN model and the uni-biometric recognition systems' performance on the face, iris (right, left), and fingerprint (right index, left index) with ROC, true prevalence curve, and AUC against FERET and Shahed face databases, iris CASIA database, as well as Shahed index fingerprint database.

The AUC using the LDA feature extraction technique in Gaussian reproducing kernel Hilbert space and angle distance classifier(Dis-Angle), as well as Mahalanobis distance classifier (Dis-L1) against FERET and Shahed databases, amounts to 0.7881 and 0.9094 respectively as represented in Figures 10(a) and 10(b).

To investigate the uni-biometric iris recognition system, several 100 CASIA database classes relative to the left and right irises are considered, from which 3 images of each iris are evaluated against the left iris database, 2 for training and 1 for testing. Additionally, from the right iris database, 4 images are considered in each class, 3 for training and 1 for testing. As for feature extraction, the Daugman and Hough transform algorithms were utilized, and some 9,600 features were extracted from the iris. Next, the five reproducing kernels (gaussian, polyplus, polynomial, linear, and hamming) were used to map the feature vectors from feature space to RKHS, through which the nonlinear relations were converted into linear ones. A further comparison of the uni-biometric iris recognition systems' performance is made in Figures 10(c) and 10(d) following their ROC curves, depicted based on Daugman and Hough transfer algorithms to extract the features of the right and left irises CASIA database, respectively. Initially, 8 Log-Gabor filters are applied at several frequencies, and some 73960 specific features are extracted for every fingerprint. As for the right and left index fingerprint databases, figures 0.8553 and 0.9593 are obtained, respectively, as the areas under the ROC curve (AUC) in the uni-biometric system.

### 5.2. Multialgorithm Iris and Multi-Instance Fingerprint Recognition Systems

Table 3 shows the AUC with the CNN model and the classification results of multialgorithm and multi-instance systems in RKHS concerning five reproducing kernels.

**Figure 11:**
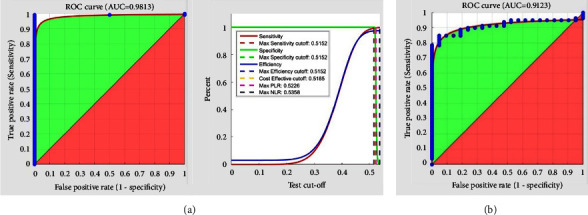
Comparison of the performance and ROC curves of (a) multialgorithm combination of right and left iris CASIA databases (combined strategy: serial rule & classifier: dis-angle) and (b) multi-instance combination of right and left index fingerprint shahed database (combining strategy).

Compared with mapping the feature space to RKHS through Gaussian and linear reproducing kernels, the recognition precision would increase to 99.07 and 100 percent, respectively, with a combined vector dimension of 180 and a reduction-based fusion dimension of 83.  Figure 11(a) compares more accurately the multialgorithm iris recognition systems' performance following their ROC curves, drawn from the application of Daugman and Hough transfer algorithms and CNN for extraction of features of left and right irises CASIA database.

Upon applying LDA and PCA in the RKHS, fingerprint features are reduced from 73960 to 150 features. When the right and left index fingerprint feature vectors are combined in the RKHS, the produced vector is inputted to the classifier. When applied for transferring the feature vectors from the feature space to RKHS, linear, and Gaussian reproducing kernels would enhance the recognition precision by 84% and, in respect of the multi-instance system, by 81%.  Figure 11(b), illustrating the ROC curve, denotes the multi-instance recognition systems' performance when the Dis-Angle classifier and CNN-based are applied to the left and right fingerprint databases. Considering the multi-instance fingerprint system, the area shown below the ROC curve (AUC = 0.9123) represents the proper performance of the system.

### 5.3. Deep Hybrid Multimodal Recognition System

Table 4 compares the recognition precision of multi-instance fingerprint and multialgorithm iris systems and hybrid multi-modal systems applying 4 approaches concerning dimensionality reduction, serial combination, parallel fusion through Quaternion, and CNN-based fusion. The deep mixed multimodal template results from a CNN-based fusion accompanied by the parallel fusion of face, combined irises, and fingerprint vectors through the Quaternion algorithm in RKHS. With the introduced hybrid multimodal system, using CNN-based fusion and parallel feature fusion through face Quaternion, combined irises, and fingerprint feature vectors using QSVD-QPCA algorithms in the RKHS, 100 percent recognition precision is achieved.

## 6. Conclusion and Future Work

Due to the richness of information available in the feature space (in terms of quality and quantity), feature-level fusion is more effective than fusion at other levels (sensor, score, and conclusion). This paper proposes a deep hybrid multimodal biometric system to obtain a robust and secure hybrid templet from the fusion of face, irises, and two left. The right index fingerprints are featured at the feature level. In one of the proposed strategies, the fusion of feature spaces based on deep learning algorithms is performed using a combination of feature vectors in in-depth and fully connected layers. The second proposed methodology was implemented based on a design involving the parallel fusion of feature spaces in the human face, combined iris and fingerprint using quaternion in the reproducing kernel Hilbert space. Using appropriate kernel functions for mapping feature vectors to the RKHS makes the nonlinear relations linear and, in other words, achieves more resolution of nonlinear relationships of biometric feature vectors in the new space. Then, in the RKHS, three feature vectors fill the three imaginary parts of the quaternion. Using the parallel fusion approach, quaternion-based algorithms extract the global and local information to constitute the quaternion fusion features based on the global and local information extracted.

Biometric systems can be evaluated using the AUC without specifying client and impostor priors or costs associated with the different errors. An AUC value of 1 indicates a perfect verifier has no false rejects and no false accepts. Verifiers that perform like random guesses have an AUC of 0.5. A verifier should perform better than a random guess, at the very least. The higher the AUC value, the better the verifier.

As for the FERET and Shahed face databases, right and left index fingerprint databases, and right and left iris CASIA databases, figures 0.7881, 0.9094, 0.8553, 0.9593, 0.8892, and 0.9593 are obtained, respectively, as the AUC in the uni-biometric system.

Also, the proposed strategies for feature-level deep fusion in the multialgorithm iris recognition system with an AUC = 0.9813 and the multi-instance fingerprint recognition system with an AUC = 0.9123, as expected.

For searching extensive databases (recognition), CNN-based and quaternion-based feature-level fusion is recommended for RKHS. Based on the results, the corresponding class of a test sample can be accurately differentiated in a secure multimodal template database without consistency errors. It is 100% accurate and performs well. Several research topics are retained for future work. One issue is how to perform analysis on other large multibiometric databases. It would be essential to obtain a robust and secure hybrid templet from the strategy of vector fusion in the feature space and repeat and evaluate again the methods which are used through one of the three mentioned processes, given that the quality of the data would be impaired and the volume would be larger. Also, another topic is to analyze the computational cost of the algorithms. [55–60].

## Figures and Tables

**Table 1:** Multiplication of basic elements.

x	1	*I*	*j*	*k*
1	1	*I*	*j*	*k*
*i*	*I*	−1	*k*	−*j*
*j*	*J*	−*k*	−1	*i*
*k*	*K*	*J*	−*i*	−1

**Table 2:** Performance parameters.

Sensitivity	TPR(True Positive Rate)=TP/TP+FN=TP/P=Recall=1 − FNR(False Negative Rate)

Specificity	TNR(True Negative Rate)=TN/TN+FP=TN/N=Selectivity=1 − FPR(False Positive Rate)

Positive likelihood ratio	Sensitivity/1 − Specificity=TPR/FPR

Negative likelihood ratio	1 − Sensitivity/Specificity=FNR/TNR

Accuracy	TP+TN/TP+FN+TN+FP=TP+TN/P+N

Precision	TP/TP+FP

**Table 3:** Comparison of the multialgorithm and multi-instance systems in RKHS, based on CNN in terms of iris and fingerprint recognition accuracy.

Multi-instance fingerprint & multialgorithm iris recognition system								
Database	Train image	Test image	Feature extraction	Dimensionality reduction		Classification	Accuracy rate (%)	AUC
Right-CASIA	324	108	Daugman	None	Dim = 9600	Dis-L1	95	0.8892
Left-CASIA	324	108	Hough	KLDA	PolyPlus (*d* = 2)	Angle	90	0.9593
Combined iris	324	108	—	KLDA	Linear	Angle	98	0.9813
Right-index shahed	400	100	Gabor	KLDA	Gaussian (*t* = 269)	Angle	53	0.8553
Left-index shahed	400	100	Gabor	KLDA	Gaussian (*t* = 269)	Angle	63	0.9593
Combined fingerprint	400	100		KLDA	Linear	Angle	70	0.9123

**Table 4:** Comparison of the results of strategies of serial combination, dimensionality reduction, parallel fusion, and CNN-based fusion.

Deep hybrid multimodal biometric recognition system					
Strategy	Bio.	Algorithm	Reproducing kernel	Dim	Accuracy rate (%)
Reproducing kernel hilbert space	Face	KLDA	Hamming	90	95
	Combined iris	Dim reduction	Linear	100	97.22
	Combined fingerprint	Dim reduction	Gaussian (*t* = 49)	99	79

CNN	Face	CNN	Softmax classifier		95
	Combined iris	CNN	Softmax classifier		98
	Combined fingerprint	CNN	Softmax classifier		89

Fusion strategy	Serial combination		Serial rule	289	84
	CNN		Softmax classifier		100
	Dimensionality reduction		Linear	50	98
	Parallel fusion	Quaternion	QPCA	120	100
			QSVD	120	100

## Data Availability

Data is available and can be provided over the emails querying directly to the author at the corresponding author (doostari@shahed.ac.ir)
